# On Muriate of Opium

**Published:** 1849-07

**Authors:** J. G. Nichol


### ARTICLE IV.

### *On Muriate of Opium.* By J. G. Nichol.

During the last ten or twelve years I have made and prescribed a solution of opium, which, I think, is not mentioned in any work on Materia Medica with which I am acquainted. I use powdered Turkey opium and water, pretty strongly acidulated with muriatic acid. I have found, by experience, that this is the best anodyne I am acquainted with. I see, by Dr. Pereira's Materia Medica, that mention is made of Dr. Porter's solution of opium in citric adid. I made and used the same sort of preparation ten years ago; but it did'nt answer. It caused a great deal of headache, and other unpleasant symptoms: moreover, it became muddy, and appeared to be decomposed; therefore, I gave up using it. I have called this preparation of mine Muriatic solution of opium, but perhaps it is not a very correct name. I may mention that I prepared solutions of opium with acetic, nitric, sulphuric, citric, tartaric, and muriatic acids, and also prescribed them, but the muriatic solution was vastly superior to any other in every respect. All of them produced *headache except muriatic.* I prefer muriate of opium to the tincture, wine, or powder of opium, and also to the muriate and acetate of morphia; in fact to any other preparation of opium. It never makes any headache but all the other preparations do.

My preparation is made according to the following formula:

Take of the best Powdered Opium,
Muriatic Acid, a a ℥. j.
Distilled Water, ℥. xx. M.

Shake this mixture very frequently every day, during fourteen days, then strain and filter. The dose is from twenty to forty drops, according to circumstances. Many of my medical friends have tried this preparation, and highly approve of it.—*Dub. Med. Press.*

